# YouTube Video Comments on Healthy Eating: Descriptive and Predictive Analysis

**DOI:** 10.2196/19618

**Published:** 2020-10-01

**Authors:** Shasha Teng, Kok Wei Khong, Saeed Pahlevan Sharif, Amr Ahmed

**Affiliations:** 1 Faculty of Business and Law Taylor's University Subang Jaya Malaysia; 2 School of Computer Science University of Nottingham Malaysia Campus Semenyih, Selangor Malaysia

**Keywords:** YouTube comments, text mining, healthy eating, clustering, structural equation modeling

## Abstract

**Background:**

Poor nutrition and food selection lead to health issues such as obesity, cardiovascular disease, diabetes, and cancer. This study of YouTube comments aims to uncover patterns of food choices and the factors driving them, in addition to exploring the sentiments of healthy eating in networked communities.

**Objective:**

The objectives of the study are to explore the determinants, motives, and barriers to healthy eating behaviors in online communities and provide insight into YouTube video commenters’ perceptions and sentiments of healthy eating through text mining techniques.

**Methods:**

This paper applied text mining techniques to identify and categorize meaningful healthy eating determinants. These determinants were then incorporated into hypothetically defined constructs that reflect their thematic and sentimental nature in order to test our proposed model using a variance-based structural equation modeling procedure.

**Results:**

With a dataset of 4654 comments extracted from YouTube videos in the context of Malaysia, we apply a text mining method to analyze the perceptions and behavior of healthy eating. There were 10 clusters identified with regard to food ingredients, food price, food choice, food portion, well-being, cooking, and culture in the concept of healthy eating. The structural equation modeling results show that clusters are positively associated with healthy eating with all *P* values less than .001, indicating a statistical significance of the study results. People hold complex and multifaceted beliefs about healthy eating in the context of YouTube videos. Fruits and vegetables are the epitome of healthy foods. Despite having a favorable perception of healthy eating, people may not purchase commonly recognized healthy food if it has a premium price. People associate healthy eating with weight concerns. Food taste, variety, and availability are identified as reasons why Malaysians cannot act on eating healthily.

**Conclusions:**

This study offers significant value to the existing literature of health-related studies by investigating the rich and diverse social media data gleaned from YouTube. This research integrated text mining analytics with predictive modeling techniques to identify thematic constructs and analyze the sentiments of healthy eating.

## Introduction

### Background

Poor nutrition and food selection lead to health issues such as obesity, cardiovascular disease, diabetes, and cancer. In order to reduce the risk of these diseases, it is important to follow healthy nutrition choices. Food selection plays a major role in a healthy lifestyle. Studies have shown that taste, cost, nutrition, convenience, pleasure, and weight control are the predicting factors for food selection [1]. This aligns with a number of dietary recommendations and public health campaigns aiming to encourage people to eat healthily.

The majority of online comments contain opinions. Social media platforms serve as a key source for obtaining large, current datasets [2]. This study focuses on YouTube comments because YouTube’s environment is multilingual, multidomain, and multicultural [2], and YouTube audiences, in turn, are international and intergenerational. In other words, the diverse nature of YouTube comments makes it a potentially valuable source of opinions on videos and the issues depicted in them [3]. In studies on health-related issues, YouTube qualifies itself as a natural candidate for researchers to study topics in the development of community and data exchange [4]. YouTube contains a large volume of comments made by video audiences. Although these comments reflect the commenters’ assumptions about food choices and health awareness [5], little is known about what people are discussing in terms of healthy eating. The aim of this study of YouTube video comments related to healthy eating is to uncover the reasons for and patterns of food choices in networked communities.

### Objectives

The objectives of the study are to explore the determinants, motives, and barriers to healthy eating behaviors in online communities and provide insight into YouTube video commenters’ perceptions and sentiments of healthy eating through text mining techniques. The study addresses the following questions: Can we identify the relevant topics and categories of healthy eating comments meaningfully? Can we interpret people’s sentiments related to healthy eating?

### Review on Healthy Eating

Healthy eating is defined as having a diet that is low in fat and high in fiber, with more fruits and vegetables in addition to balance and variety [6]. Bisogni and colleagues [7] conducted a systematic review of past qualitative research studies to understand how people interpreted healthy eating. Their research revealed that people associated healthy eating with fruits and vegetables, animal-based products, safe food, functional food, general nutrients, fiber, vitamins and minerals, balance, variety, and moderation, with descriptors such as fat, natural, organic, homemade, and so on. People discussed healthy eating in terms of foods, nutrients, or other components. Another study focused on group interviews with adolescents from high school. The results showed that young people considered healthy eating to be eating the right types of food, and they went ahead to name specific foods, such as fruit, salad, and yogurt [6]. They also included carbohydrate-rich foods (eg, rice and pasta), lean meat (baked chicken in particular), and tofu. Home-grown vegetables, greens, corn, and celery were mentioned during the interviews. The adolescents shared their understanding of unhealthy eating by naming specific foods, such as fast food, candy, soda pop, and chips [6]. Lu and Gursoy [8] examined the influencing factor of organic food on consumer decision making. The results indicated that healthy food described not only a low-fat or low-calorie diet but also a meal loaded with nutrients and quality ingredients (eg, organic food).

In the 20th century, the Mediterranean diet pyramid was presented in the study by Willett and colleagues [9]. This diet is characterized by abundant plant food, fresh fruits daily, olive oil as the principal source of fat, dairy products (in particular cheese and yogurt), fish and poultry consumed in moderate amounts and red meat consumed in low amounts, and wine consumed in low or moderate amounts during meals [9]. The study exemplified the abundance of plant food with couscous, vegetables, and legumes in North Africa, pasta, rice, and vegetables in South Europe, and bulgur, rice and vegetables, chickpeas, and other beans in the Eastern Mediterranean regions. Olive oil is low in saturated fat, and it is a major source of fat in the Mediterranean diet. Dairy products are included in this diet from animals such as goats, sheep, cows, and buffalo. These products are consumed in small to moderate portions. Compared with the high intake of meat in the Western diet, the Mediterranean diet recommends red meat in low amounts, along with moderate consumption of fish and eggs. A healthy lifestyle would require regular physical activity and sufficient relaxation. Carefully prepared delicious food and sharing food with family and friends stimulated the enjoyment of a healthy Mediterranean diet. Apart from academic research, the public health sector in the United States promoted healthy eating by releasing the Healthy Eating Index (HEI) in 1995. The HEI-2005 dietary guidelines advise people to eat fruit instead of fruit juice, to eat dark green and orange vegetables, and to eat legumes. Milk, meat, and beans are included in the index. HEI-2005 assesses the intake of food and nutrients from a density basis, which is calculated as a ratio to energy intake. The 1200- to 2400-calorie weekly pattern meets the recommended nutrient intake, including a 1000-calorie weekly intake of dark green and orange vegetables and legumes. HEI-2005 includes guidance on sodium and saturated fat intake as well. The dietary guidelines encourage people to consume low-fat food free of added sugar and avoid solid fat and alcoholic beverages [10].

Many studies have explored the determinants of healthy eating behavior. In terms of food characteristics, Lu and Gursoy [8] examined food quality from the diners’ decision-making aspect. The results showed that food presentation, the presence of nutritious ingredients, taste, and freshness are the critical factors that influence the diners’ attitudes toward visiting certain restaurants. Examining a review of academic papers focused on children and youths, Taylor et al [11] revealed that children’s food preferences are an important predictor of healthy eating. They believe that a lack of nutrient knowledge can lead to poor food choices [11]. Differences in the determinants of eating behaviors were found in boys and girls. Deshpande et al [1] investigated factors influencing college students’ healthy eating habits and found that female students tend to eat more fatty food than male students. They also concluded that female and male students are motivated to eat a healthy diet by different factors. This result is consistent with the study by Croll et al [6]. Girls eat healthy food in order to have a better appearance, whereas boys eat healthily to maintain energy. Food price is considered to be an economic determinant of healthy eating. As agreed by Taylor and colleagues [11], the most common consideration in reference to food choice is food price. In other words, having a low income leads to selecting food that is high in sugar and fat as these foods are cheaper. This also means that people have low-quality meals [8]. Moreover, another study found that the food price is higher for healthy food than any other food in grocery stores. These grocery stores appear to have more positive reviews regarding the availability of quality healthy food [12]. Parenting styles and one’s peers are regarded as the social determinants of healthy eating. Students off campus choose different foods from students living on campus [1]. The physical environment determinants include food portion size and school environment. Studies have been carried out to implement environmental interventions in reference to healthy eating [1,13]. This provides a chance to eliminate or weaken an unhealthy environment so it is easier for people to engage in health-enhancing behavior. In addition, environmental interventions promote role models and social support for the members of the community.

Studies have investigated motives influencing healthy eating behavior as well and have indicated that a healthy appearance, positive feelings, and preventing disease are the factors behind college students having a healthy diet [1]. Similar findings were included in a study by Shepherd et al [14]. Previous work revealed that weight management is influenced by healthy eating [15]. A number of barriers to healthy eating were examined as well. Shepherd et al [14] identified that the poor availability of healthy food at school, a lack of healthy eating information from teachers and friends, the students’ preferences for fast food, and expensive healthy food are barrier factors [14]. Deshpande et al [1] included time sufficiency, convenience, and a higher perception of stress and low self-esteem as student barriers to healthy eating. It is widely known that a lack of nutrition knowledge is attributed to barriers to healthy eating.

### YouTube Video Comments

We reviewed the literature related to YouTube video comments and found that a number of studies have categorized YouTube comments through various approaches given the complexity of YouTube comment characteristics. Madden et al [16] applied a classification scheme that covers impressions, advice, opinions, and comments in terms of YouTube use. This scheme reveals differences in YouTube video commenting behavior regarding different genres. Their study clustered 10 detailed categories of YouTube comments, including information, advice, impression, opinion, responses from previous comments, expression of personal feelings, general conversation, site process, video content description, and nonresponse messages [16]. Another study categorized YouTube comments related to health issues into different groups: self-disclosure comments, feedback for the video uploader, factual in nature, help-related messages, and so on [17]. Wendt et al [18] studied significant differences in user perceptions of viral stealth videos and product advertising videos. Topic, pragmatics, and sentiment are the three main categories of YouTube videos. It is recognized that comments are generally positive toward these videos. Madden et al [16] explored the nature of YouTube comments to understand perceptions of the utility of YouTube videos by developing a category scheme. Their scheme included questions, responses, feedback (general), feedback (positive/agree), feedback (negative/disagree), appreciation/acknowledgment, giving personal information/situation, personal action, spam/promotion, and unclassifiable messages [19]. Besides categorizing YouTube comments, several studies explored the characteristics of YouTube videos, such as video type, viewer engagement level, technique use level, and message level [20]. Ferchaud et al [21] established categories from video content feature and production feature aspects in order to explore parasocial attributes and YouTube personalities [21]. Zhang et al [22] demonstrated that statistical evidence, narrative, humor, fear image, and peer influence are important types of video message appeal that stimulate more views, likes, and comments on YouTube videos related to healthy eating.

### Text Analytics

In studies of health research, surveys and consultations with patients have the advantage of involving a large sample. Conducting these surveys may potentially prevent geographical dependence, yet the number of answers is restricted in questionnaires, which can reduce the chances of capturing deep input [23]. Additionally, low response rates and the difficulty of obtaining an essential understanding of sensitive issues are other disadvantages of traditional survey methods [24]. In contrast, interviews and focus groups can offer the possibility of obtaining detailed information [23]. However, given the relatively small sample size, these methods may overlook important aspects of the interviewees’ opinions. Long and diffused answers generated from interviews make them difficult to understand or analyze through a traditional approach [25]. Bicquelet [23] applied the text mining technique to gather online data in order to understand patient needs. This increasingly popular social research technique has been adapted to analyze levels of interest in a topic or a time series analysis of trends in interest [3]. Text mining allows researchers to process data from a large number of web pages. In recent years, this automated analysis has quickly and effectively interpreted large bodies of extracted online data, thus promising valuable insights across different research areas [23].

Feldman and Sanger [26] defined text mining as extracting the corpus of the data and identifying patterns and relationships in the textual data automatically. In other words, it is a process of extracting meaningful information pieces and subsequently applying algorithms to the extracted data and deriving structured variables for further analysis [27]. Text mining focuses on areas such as retrieving information, classifying and clustering texts, natural language processing, and so on. With the growing availability and popularity of social media platforms, sentiment analysis has emerged as a novel research area within text analytics in recent years [26,28]. Traditionally, sentiment analysis is about whether someone has a positive, neutral, or negative opinion toward something. In the context of social media, sentiment analysis aims to determine the opinion polarity of online reviews made by internet users toward products and services [28]. As an automated information obtaining technique, sentiment analysis can find the hidden patterns embedded in reviews, blogs, and tweets. It is extremely valuable to obtain this knowledge, as the opinions expressed within social media networks are genuine [29]. We observed that these affective opinions are easily understood, and they become the basis of decision making. Although sentiment analysis within text analytics has been applied in the political science, marketing, and consumer behavior fields, it has rarely been used to extract meaningful information from online data on health-related issues.

Researchers have applied new methods to analyze the contents and sentiments of YouTube comments. For example, Severyn et al [2] proposed a shallow syntactic structure to test and predict comment types and their polarity in English and Italian. They believe that this structure outperforms the traditional approach, such as the bag-of-words model, in terms of mining opinions. HarVis was introduced by Ahmad et al [4] as a tool to facilitate YouTube content acquisition, data processing, and visualization on any topic. Thelwall [3] developed comment term frequency comparison in order to investigate YouTube video topics as a novel social media analytics approach.

Sentiment analysis is a trending topic among YouTube video comments studies. One study investigated co-commenting behavior on K-pop videos by analyzing the weighted frequency and weighted sentiment scores of the co-comments. The results indicated that a large number of co-comments are positive, which impacted the co-commenting behavior in the K-pop video community [30]. Another study used SentiWordNet to analyze the comment ratings on the YouTube video platform. The findings showed that community feedback with term features is an indicator of the community acceptance of the comments [31]. This study will apply text mining techniques to extract YouTube healthy eating video comments and interpret them in order to understand people’s perceptions and sentiments related to healthy eating.

## Methods

### Video and Comments Selection

This study analyzed comments scraped from YouTube related to healthy eating videos to investigate the perceptions and sentiments of YouTube audiences. It is set in Malaysia, where 11% of Malaysians have diabetes, the highest rate of incidence in Asia and one of the highest in the world. We are motivated to understand the reasons for this health care predicament. We thus intend to examine healthy eating behaviors in this country. Given the enormous volume of YouTube video comments, we were able to gather data concentrated on healthy eating. Hence, a systematic search was conducted to select comments for analysis. The key phrase “healthy eating in Malaysia” was entered into the YouTube search engine. With the help of the filter function, videos were selected and ranked by relevance in descending order. The criteria for selecting videos in this study are the relevance of YouTube video contents of healthy eating in Malaysia, the number of each video’s views, and the number of comments generated by commenters. The authors watched the videos from the first 5 consecutive results pages in order to select all of the relevant videos. They progressed through the rest of the list until the searched videos became totally unrelated. Following the steps by Meldrum et al [19], videos pertaining to non-Malaysian food, videos in languages other than English, videos targeting specific audiences, and videos that had disabled the commenting feature were excluded. Additionally, videos with fewer than 50 comments were excluded. In total, 10 videos remained after the authors independently screened and recorded the titles and links of the qualified videos. Replies to the existing comments and comments left by the video’s creators were included as this content analysis focused on all of the community members’ opinions on healthy eating. Overall, 5756 comments and replies posted under the 10 videos were scraped from YouTube [32] and transferred to an Excel spreadsheet (Microsoft Corporation). The authors read through the dataset and removed data entries such as #NAME#, external links, and advertisements. This data cleansing process returned 4654 data entries for the content analysis. Table 1 summarizes the videos included in this study. Similar to previous studies [17,19,33], as this study involved anonymous, publicly available data, it was deemed as exempt by the human ethics committee of Taylor’s University.

**Table 1:** Overview of selected YouTube videos.

Title	Views, n	Comments, n
How I Eat Healthy on a Low Budget! (Cheap & Clean) [34]	1,197,933	1815
Must Eat “Free” Foods to Stay Slim [35]	1,152,241	745
Dieting Mistakes—Why You're Not Losing Weight! [36]	1,034,300	872
My EAT CLEAN Meal Plan (Full Recipes) [37]	577,141	373
It’s Tough Dieting in Malaysia [38]	534,511	877
Healthy Foods That Can Make You GAIN WEIGHT [39]	436,489	668
Budget Meal Prep As a College Student! [40]	127,003	103
The Ultimate MALAYSIAN Healthy Food Swaps — Eat This. Not That [41]	52,418	196
We Try Eating Healthy for 2 Weeks — SAYS CubaTry [42]	39,959	53
What I Eat in a Day in Malaysia (video currently inaccessible)	25,552	54

### Data Analysis

This study used a computer-assisted content analysis package called Text Miner version 9.4 (SAS Institute Inc) to analyze the comments from selected YouTube videos related to healthy eating. This software combines textual and statistical analysis by focusing on the frequency of words in a corpus. For example, homogeneous subsets of words are identified on their lexical basis. At the initial stage, the SAS software extracts, stems, and filters words, which is called text parsing in the data analysis process. After the YouTube comments are tokenized, the identified terms are listed in terms of word frequency (see Table 2). During the text parsing node, parts of speech such as prepositions, pronouns, and auxiliary verbs are ignored. Text parsing facilitates the systematic splitting of texts into sets of controllable terms in the text corpus. By eliminating extraneous terms, the text filter node keeps the relevant and meaningful terms that are shown by their frequency of occurrence in the dataset. The increased signal-to-noise ratio enhances the quality measure of the dataset [43]. The two processes led to a robust set of relevant terms for further analysis via the term frequency–inverse document frequency algorithm (see Multimedia Appendix 1). This algorithm retains terms that are deemed relevant to the study like “healthy,” “vegan,” “weight,” “workout,” etc, while removing stopwords like “the,” “a,” “on,” and “in,” etc.

**Table 2:** Excerpts of the term frequency document.

Term	attrstring^a^	Weight	Frequency	Keep
+^b^ eat	Alpha	0.2600599	610	Y
+ video	Alpha	0.2509651	444	Y
+ food	Alpha	0.2883606	486	Y
+ good	Alpha	0.2775832	368	Y
+ love	Alpha	0.3065190	282	Y
+ healthy	Alpha	0.3331854	271	Y
+ people	Alpha	0.3584516	245	Y
+ weight	Alpha	0.3598992	238	Y
+ know	Alpha	0.3606262	200	Y
joanna	Alpha	0.3550649	176	Y
+ diet	Alpha	0.3694043	213	Y
+ egg	Alpha	0.3739284	241	Y
+ buy	Alpha	0.3863306	220	Y
+ great	Alpha	0.3680809	172	Y
+ live	Alpha	0.3922955	162	Y
+ lose	Alpha	0.3916902	153	Y
+ time	Alpha	0.4046275	138	Y
+ tip	Alpha	0.3997993	124	Y

^a^attrstring: in the Alpha attribute, numeric characters are stored as a string.

^b^+ depicts parent term.

Text clustering was then performed on the term frequency document by successively splitting terms into mutually exclusive groups. The process was conducted using the latent semantic indexing procedure to group terms into higher order semantic structures [44]. These groups were constructed based on similar forms (or words). The latent semantic indexing procedure in this study used the singular value decomposition (SVD) algorithm (see Multimedia Appendix 2) to reduce the terms in Table 2 into a set of manageable clusters. Thus, the topics/themes are as revealed in Table 3. The results of the clustering via SVD on the term frequency document were set to generate enough SVD dimensions (k) for further analysis. The greater the number of dimensions (k), the higher the resolution to the term frequency clustered. In this study, k was set to 50, which was the default setting in the SAS software. The k-dimensional subspace was then generated from the cluster analysis via SVD. The cluster frequency root mean square standard deviation shown in Table 3 shows that the derived clusters via SVD were well manifested and optimal as the values are close to 0.

**Table 3:** Summary of the text clusters.

Cluster ID	Cluster description	n (%)	RMS^a^ SD
A12	+^b^love awesome lol +true joanna ming +diet omg +work malaysia	416 (15.00)	0.103737
A18	+video +love joanna videos soh informative helpful ur +help +watch +channel +weight	369 (13.00)	0.125160
A11	+food +healthy +meat foods +protein +animal +eat +cheap +vegan +know veggies afford	322 (11.99)	0.127740
A17	+people eggs caged chickens animals understand dont +comment +vegan +bad +stop +chicken	341 (11.99)	0.133414
A13	+fat +budget +buy +low calories expensive +organic water +healthy +want foods	287 (9.99)	0.134404
A25	+good best better first advice +girl +body +smart +fat +gain eating “a lot of”	247 (9.00)	0.125705
A15	+great tips +video “great video” +rice “great tips” brown +tip awesome +white joanna looking	234 (8.01)	0.118946
A14	+true +malaysian +food malaysia +organic nasi +diet foods mamak lemak +exercise lah	195 (7.00)	0.124926
A19	butter +peanut +story +sugar watching +time coconut +avocado based +small +old +channel	183 (7.00)	0.132021
A22	+weight +lose +workout +week kg lost frozen +fresh +gym veggies +hot +buy	198 (7.00)	0.130526

^a^RMS: root mean square.

^b^+ depicts parent term.

## Results

### Cluster

This section describes the results of applying text mining methods to the topic of healthy eating in YouTube videos. Table 3 presents the major themes, sometimes called dimensions, that emerged from the data analysis. There were 10 clusters identified with regard to food ingredients, food price, food choice, food portion, well-being, cooking, and culture in the concept of healthy eating. These clusters are reflected in the most frequent key terms used by the YouTube commenters. The key terms were automatically selected on the basis of their occurrence and co-occurrence. The authors read through the key terms and sentences extracted from YouTube videos in order to make sense of the clusters categorized by the software.

### Cluster A12: +love awesome lol +true joanna ming +diet omg +work malaysia

This cluster is mentioned in 15.00% (698/4654) of comments related to healthy eating. Many comments were related to the commenters’ opinions of YouTube videos in the context of healthy eating in Malaysia. They expressed their feelings and gratitude toward the video creators by commenting “love,” “awesome,” and “thank you for sharing.”

### Cluster A18: +video +love joanna videos soh informative helpful ur +help +watch +channel +weight

In total, 13.00% (605/4654) of comments gleaned from the YouTube videos contribute to cluster A18. Similar to cluster A12, people expressed their point of view on the usefulness of YouTube videos. Key terms were “informative” and “helpful.” A typical example of this cluster follows:

Thanks so much for this video, it is very informative!

### Cluster A11: +food +healthy +meat foods +protein +animal +eat +cheap +vegan +know veggies afford

This cluster demonstrated that animal rights were mentioned. People who go vegan tend to want a healthy lifestyle. Others believe humans are omnivores, but they are concerned with animal cruelty during the production process. A representative comment follows:

Humans are omnivore beings, there’s nothing wrong or evil with eating meat. The way they are incarcerated and killed is made the way it is to make it cheaper and more profitable for the companies who produce them. It might not be the kindest way to deal with living beings, but it does make the process cheaper, taking food for more people’s tables.

### Cluster A17: +people eggs caged chickens animals understand don’t +comment +vegan +bad +stop +chicken

This cluster was mentioned in 11.99% (558/4654) of comments related to healthy eating. A large number of YouTube commenters buy free-range chicken eggs to support animal welfare, whereas others cannot afford cage-free eggs as caged chicken eggs are cheaper. The emphasis is on food price. The argument of animal rights divided the commenters into two groups. Some are concerned about animal cruelty in relation to caged chickens. Others expressed their understanding of animal rights but explained that they cannot afford healthy eggs that are produced by free-range local farms. One of the most representative sentences under cluster A17 follows:

All I hear in the comments is “privilege!” I am a supporter of cruelty free products and natural ingredients but we have to be realistic about how the world works. The poorer you are (at least in the states) the higher risk you have for being unhealthy and attracting diseases. There are literally people who have no access to fresh foods known as “food deserts” and live off of canned and processed food because of their socioeconomic status.

### Cluster A13: +fat +budget +buy +low calories expensive +organic water +healthy +want foods

This cluster is mentioned in 9.99% (465/4654) of YouTube comments. The commenters love the video, and they can relate to the video personally. Most people agree that we need to eat organic food. Some said that they cannot afford it. A number of people think that organic is simply a marketing term used by industries to make profits.

Yes, organic food is a lot more expensive and not affordable for everyone. If you like buying organic food and your opinion is that it is better then you should. Just know that it is not more nutritious; the taste is 100% objective; depending on where you get your organic food from it may not be sustainable and may use chemicals you don’t normally think of as organic; you are also paying about 30% more for no reason other then the fact they put an organic sticker on it.

### Cluster A25: +good best better first advice +girl +body +smart +fat +gain eating “a lot of”

This cluster deals with people’s perception of YouTube videos, which are “good,” “better,” and “smart.”

### Cluster A15: +great tips +video “great video” +rice “great tips” brown +tip awesome +white joanna looking

A number of comments contained the commenters’ opinions of YouTube videos and video contents. As shown in this cluster, the key terms are “great” and “great tips.” People expressed their gratitude for the method of mixing brown rice and white rice, as suggested by YouTube creator Joanna. Many people support cooking and preparing meals at home. Some said that fast food is cheaper than homemade meals.

This white rice/brown rice mix tip is awesome!! Making homemade energy bars is a great option. You know precisely what went in it.

### Cluster A14: +true +malaysian +food malaysia +organic nasi +diet foods mamak lemak +exercise lah

This cluster deals with the perceptions of Malaysian food in terms of healthy eating. Fat, sugar, and oils are most frequently mentioned in the YouTube comments. People believe that Malaysian food is high in calories and carbohydrates. People associated sugar with Malaysian food as well. In addition, it is tough to diet in Malaysia. Mamak (comfort food) restaurants are open 24/7, making it hard to resist Malaysian food. In other words, the cultural factor becomes a barrier to healthy eating in Malaysia. A typical comment follows:

Woww im so proud =))) I’m trying to follow your vidoes to eat healthier and to get up and exercise... You know how hard it is being a Malaysian because we love food so much ;)

### Cluster A19: butter +peanut +story +sugar watching +time coconut +avocado based +small +old +channel

In this cluster, people associated healthy eating with food choices. Similar to a previous study [14], fruits are the most commonly mentioned healthy food. Key terms are coconut and avocado. An example follows:

I eat an avocado maybe one time a week as a meal with a piece of toast and an egg. I think avocados are great, just a once in a while thing tho.

### Cluster A22: +weight +lose +workout +week kg lost frozen +fresh +gym veggies +hot +buy

This cluster illustrates the relationship between healthy eating and weight management. Commenters expressed their points of view on the challenges and barriers to healthy eating. The focus is that Malaysians find it very hard to eat on a diet; therefore, it is difficult to experience weight loss. Reasons such as delicious food choices are included. Exercising does not keep the body in shape as people go to Mamak restaurants after their workout. An example of a cluster A22 comment follows:

Before I came to malaysia, I did my diet as usual, but when i’m in malaysia I totally ruin my diet. Can’t resist mee goreng mamak and many malaysian foods.

This cluster also deals with food-storing methods. People express their views on frozen vegetables and fresh vegetables. A lot of people believe that frozen vegetables contain more nutrients than fresh vegetables. A typical comment follows:

Nutrients may be lost if frozen vegetables are slowly defrosted. i have always been told by nutritionists that frozen is healthier than fresh.

A significant degree of convergence was found between the different clusters. This suggests that YouTube comments are likely to fall into more than one category. For example, food price was mentioned in clusters A11 and A13. Cultural factors were discussed in clusters A12 and A14. Food choices and ingredients related to healthy eating were included in clusters A11, A17, and A19. In general, the key terms related to selected YouTube videos contained positive sentiments such as “good” (mentioned 326 times), “great” (257 times), “love” (423 times), and “keep up with the good work” (19 times). Attitudes toward healthy eating are generally positive, as these clusters indicate. These results showed that research question 2 with regard to interpreting the sentiments of healthy eating comments has been addressed.

Based on Table 3, each cluster was manifested inside the derived subspace (see the root mean square standard deviation); 7 clusters were plotted on a Cartesian coordinate system, and 3 clusters (A12, A18, and A25) mainly related to the usefulness and favorability of videos were not considered for further analysis. In this Cartesian coordinate system, the distance between the clusters was the space generated during the SVD procedure. Clusters with shorter distances between them depict closer relationships within their semantic structures and vice versa. The results of the factor analysis showed that the data were appropriate for this study, given the Kaiser-Meyer-Olkin measure of sampling adequacy value of .879 [45]. A Bartlett test of sphericity was significant (*P*<.001). Principal component analysis revealed that one factor was extracted and it accounted for 60.749% of the total variance. The Cronbach alpha value for the 7 items was .878, indicating a satisfactory level of reliability [46]. The *t* values in the results indicate that the critical ratios for the parameter estimates were significant at a .05 level. This result shows that convergent validity and the unidimensionality of each item met the satisfactory requirements [47]. Several model fit measures were employed to assess the model’s overall goodness of fit. The results in Table 4 showed an adequate model fit, indicating that the model was acceptable [45].

**Figure 1:**
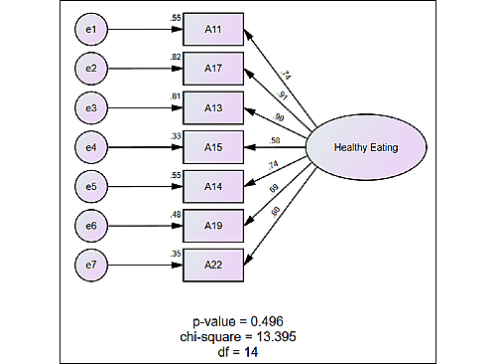
Confirmatory factor analysis results on healthy eating.

Subsequently, the confirmatory factor analysis test was conducted on the clusters to examine the relationships of the clusters derived from the unstructured data pertaining to healthy eating. The measurement model was used to test the validity and reliability of the healthy eating determinants in this study. Confirmatory factor analysis was performed using SPSS Amos version 20 for Windows (IBM Corporation), and the results are shown in Figure 1, indicating that all of the healthy eating determinants have positive relationships with healthy eating. Table 5 summarizes the associations of the determinants in the measurement model.

**Table 4:** Goodness-of-fit indices.

SEM^a^ indicators	Criteria	Results
Chi-square	—	13.395
Chi-square/degree of freedom ratio	<2	0.957
*P* value	>.05	.496
GFI^b^	>0.9	0.926
CFI^c^	>0.9	1.000
NFI^d^	>0.9	0.933
RMR^e^	close to 0	0.000
RMSEA^f^	<0.08	0.000

^a^SEM: structural equation modeling.

^b^GFI: goodness-of-fit index.

^c^CFI: comparative fit index.

^d^NFI: normed fit index.

^e^RMR: root mean square residual.

^f^RMSEA: root mean square error of approximation.

**Table 5:** Summary of the structural equation modeling results.

Path	Estimate	SE^a^	CR^b^	*P* value
A11<---Healthy eating	1.000	—	—	—
A17<---Healthy eating	0.926	0.144	6.443	<.001
A13<---Healthy eating	0.864	0.135	6.388	<.001
A15<---Healthy eating	0.763	0.191	3.987	<.001
A14<---Healthy eating	0.920	0.177	5.201	<.001
A19<---Healthy eating	0.772	0.160	4.828	<.001
A22<---Healthy eating	0.728	0.177	4.120	<.001

^a^SE: standard error.

^b^CR: critical ratio.

## Discussion

### Principal Findings

This study is one of the first attempts to identify the perceptions of healthy eating by analyzing a large corpus of data extracted from YouTube videos. Using content and sentiment analysis assisted by text analytics software, this study uncovered thematic clusters that indicate how people interpret healthy eating and what their eating behaviors are. Food choice, food price, cooking method, cultural influence, and weight management are identified as semantic clusters. A principal finding of this study is that people hold complex and multifaceted beliefs about healthy eating in the context of YouTube videos. As suggested by Bisogni and colleagues [7], a set of beliefs may not be judged as correct or incorrect according to the scientists’ standards. People also have different interests in the context of healthy eating, and they vary in their ways of dealing with changing information.

The concept of healthy eating is fairly consistent with the other studies’ findings. That is, healthy eating is always described in terms of foods, namely fruits and vegetables (clusters A19 and A22). It seems that fruits and vegetables are the epitome of healthy foods. Healthy eating is regarded as eating more fruits and vegetables, having a balanced diet, and consuming a variety of foods. People should limit their intake of high-caloric foods and avoid processed food [6]. Despite similarities with the previous studies, this study found that commenters are less likely to describe healthy eating directly, such as through nutrition. One possible explanation can be related to the nature of commenting on YouTube videos. The video creators mention specific food, and the viewers’ immediate reaction would be to the food itself; hence the comments are intuitively related to the food mentioned in the videos.

This study has meaningfully categorized the determinants of healthy eating from both individual and collective aspects. A particularly interesting and potentially important insight from this study is that people’s perceptions of healthy eating comprise social, economic, and physical well-being and moral factors (clusters A17 and A22). Free-range chicken eggs and caged chicken eggs are the most mentioned key terms in cluster A17. Animals deserve to live their lives free from suffering and exploitation. People who support animal rights buy free-range eggs. However, socioeconomically disadvantaged people can afford caged eggs only. As also seen in the study by Lu and Gursoy [8], the financial restriction factor lessens an individual’s control over conducting a given behavior. In other words, despite a favorable perception of healthy eating, people may not purchase commonly recognized healthy food for a premium price. The study results indicated that food price as an economic determinant influences people’s perceptions of healthy eating. More negative sentiments are expressed about healthy food prices. Weight concerns are associated with healthy eating. It is not surprising to see that people understand the importance of healthy eating with regard to physical well-being. This study has illustrated the priority of healthy eating as being related to the benefits of this behavior, such as weight loss, better appearance, and improved quality of life. Another key determinant identified in this study is the environmental factor. Studies have revealed that food service menu plans and the food available on campus, in addition to food stores and restaurants, contribute to a healthy lifestyle [13]. The physical environment, including home, school, and fast food establishments, plays an important part in influencing young people’s eating behaviors [11]. Our study bears a close resemblance to these two studies. The key terms from cluster A15 indicated that people associated homemade food with healthy food. College students tend to prepare their food, and they avoid fast food sold on campus. One cultural factor was identified in this study. Consistent with the study findings of Bisogni et al [7], resources and environment prevented people from eating healthily. The study identified the reasons why Malaysians cannot always act on eating healthily, such as food taste, variety, and availability. In addition, the structural equation modeling results provided valuable insights into understanding the determinants that affect healthy eating practices.

### Implications

This study contributes to the understanding of healthy eating behavior by examining a large volume of online data obtained from YouTube videos and fills the gap in previous research regarding healthy eating in the context of social media. It provides a comprehensive picture of healthy eating. The holistic view includes the perceptions, determinant factors, and benefits and barriers to healthy eating. This study highlights the fit of using content analysis while analyzing healthy eating-related YouTube video comments. Quantitative approaches are restricted to measuring aspects, like popularity [18]. This qualitative approach allows researchers to have an in-depth understanding of how people interpret and disseminate information regarding healthy eating. In addition, YouTube video comments are generally genuine personal reactions made by the commenters. Compared with the socially desirable answers obtained from surveys/focus groups, this social media data produces theoretically and empirically sound results [48]. Moreover, in comparison with the traditional sample size and response rate, this study offers significant value to the existing literature of health-related studies by investigating the rich and diverse social media data gathered from YouTube. This study sheds light on the understanding of the underlying social structure and dynamics in the context of healthy eating. People from socioeconomically affluent communities have the availability of healthy food from local farms. Some people choose to buy caged chicken eggs even though they know that it is morally wrong. This study offers a detailed picture of the impact of social and economic factors on healthy eating.

This study may assist public health professionals in making effective public health campaigns. Transcending geographical and regulatory boundaries, the internet provides the public with a source of reference material [23,49,50]. This study found that the distinction between experience and expertise is blurred with regard to healthy eating comments. Therefore, it is imperative for health professionals to use social media platforms to disseminate accurate and credible health information to lay audiences. In other words, public health professionals work on posting and replying to conversations when appropriate through comments and sharing materials with members of the social media community. Moreover, the salient findings of this study can provide nutrition educators and health professionals with meaningful and useful ideas. These professionals are advised to deliver nutrition information on calories, macronutrients (fat, protein, and carbohydrate), and micronutrients (vitamins and minerals) to the public in a concise manner. Public health campaigns could be employed to promote healthy eating by establishing appropriate interventions tailored to the targeted audience. For instance, providing affordable and appealing healthy food in schools may serve to increase the students’ willingness to eat healthily. Sustainable diets may be an alternative to promoting public health with various governmental and social support.

### Limitation and Future Studies

Some study limitations should be noted. The sample of YouTube videos was selected based on relevance and the number of views and comments. The authors conducted informal searches for videos related to healthy eating during the sampling; however, using limited key phrases when searching does not guarantee a complete and representative sample of YouTube videos. Further investigation using more videos and comments may reveal new findings related to healthy eating.

The study context of healthy eating in Malaysia is another limitation. Examining the comments from other cultures would increase the generalizability of the study. The sampling bias of YouTube video comments cannot be avoided as these comments are broad but less focused. In addition, it may be beneficial if future studies made a comparison of online data from different social media platforms regarding similar topics. With the help of text analytics software, the study identified several thematic clusters. The authors selected each category after screening the corpus of the data. Such a classification can ignore some of the significant variations within each cluster. Future research is encouraged to study more comments in order to explore new categories and subcategories of healthy eating. This study did not examine the number of likes, which is an important parameter, leaving space for future research. The authors consider this study and concurrent research as a first step to understanding the perceptions of healthy eating, not as the end of the discussion.

### Conclusion

There has been an increasing recognition that healthy food intake and balanced eating patterns play an important role in health and disease prevention. This study conducted an in-depth content analysis of YouTube comments. The text analytics method was employed as it is useful for discussing and exploring large-scale YouTube-specific phenomenon. This study provides 10 main categories related to healthy eating, including food choice, food price, cooking method, cultural influence, weight management, and so on. Structural equation modeling revealed there to be significant relationships between these categories and healthy eating. Through the lens of text analytics and sentiment analysis, our results suggest that people have a largely positive attitude toward healthy eating. This study contributes to the perceptions of healthy eating by categorizing the determinants, benefits, and barriers to healthy eating such as food choice, food price, weight management, and cultural influence. Future study is necessary to explore more of the categories, subcategories, and underlying concepts of healthy eating. The findings of this study provide support for public health professionals to allow them to better implement effective health promotion campaigns.

## Appendix

Multimedia Appendix 1 Term frequency–inverse document frequency algorithm.

Multimedia Appendix 2 Singular value decomposition algorithm.

## Abbreviations

HEI: Healthy Eating Index
SVD: singular value decomposition
