# Supplementary material for: YouTube Video Comments on Healthy Eating: Descriptive and Predictive Analysis
Source: JMIR Public Health Surveill. 2020 Oct 1;6(4):e19618. doi: 10.2196/19618 (PMC7563625; doi:10.2196/19618)
Supplement: Multimedia Appendix 1 [file publichealth_v6i4e19618_app1.pdf]

Multimedia Appendix 1: Term Frequency – Inverse Document Frequency (TF-IDF) algorithm

The TF-IDF algorithm can be notated as follows:

$$W_{td} = tf_{td} \times \log(\frac{N}{df_t}) \qquad (1)$$

Where N depicts the total number of documents in the text corpus, W_{td} depicts the weight on the term $_t$ in document $_d$, tf depicts the frequency of the terms $_t$ in the document $_d$, and df depicts the frequency of documents with the term $_t$.
