# Supplementary material for: YouTube Video Comments on Healthy Eating: Descriptive and Predictive Analysis
Source: JMIR Public Health Surveill. 2020 Oct 1;6(4):e19618. doi: 10.2196/19618 (PMC7563625; doi:10.2196/19618)
Supplement: Multimedia Appendix 2 [file publichealth_v6i4e19618_app2.pdf]

Multimedia Appendix 2: Singular Value Decomposition (SVD) algorithm

The SVD algorithm can be notated as:

$$A = U\sum V^T \qquad (2)$$

Where A depicts the decomposed matrix of m x n. U depicts the m x m matrix, $\sum$ depicts the singular value of A, V^T depicts is the matrix of n x n.
